# Rigour and Rapport: a qualitative study of parents’ and professionals’ experiences of joint agency infant death investigation

**DOI:** 10.1186/s12887-017-0803-2

**Published:** 2017-02-07

**Authors:** Joanna Garstang, Frances Griffiths, Peter Sidebotham

**Affiliations:** 1 Division of Mental Health and Wellbeing, Warwick Medical School, University of Warwick, Coventry, CV7 4AL UK; 2 Division of Health Sciences, Warwick Medical School, University of Warwick, Coventry, CV7 4AL UK

**Keywords:** Child Death Review, Bereaved parents, Sudden Unexpected Death in Infancy (SUDI), Sudden Infant Death Syndrome (SIDS), Physician-parent communication, Inter-professional working

## Abstract

**Background:**

In many countries there are now detailed Child Death Review (CDR) processes following unexpected child deaths. CDR can lead to a fuller understanding of the causes for each child’s death but this potentially intrusive process may increase the distress of bereaved families. In England, a joint agency approach (JAA) is used where police, healthcare and social services investigate sudden child deaths together and a key part of this is the joint home visit (JHV) where specialist police and paediatricians visit the home with the parents to view the scene of death. This study aimed to learn of bereaved parents’ experiences of JAA investigation following Sudden Unexpected Death in Infancy (SUDI).

**Methods:**

This was a qualitative study of joint agency investigation of SUDI by specialist police, healthcare and social services including case note analysis, parental questionnaires, and in-depth interviews with parents and professionals. Families were recruited at the conclusion of the JAA. Data were analysed using a Framework Approach.

**Results:**

21/113 eligible families and 26 professionals participated giving theoretical saturation of data. There was an inherent conflict for professionals trying to both investigate deaths thoroughly as well as support families. Bereaved parents appreciated the JAA especially for the information it provided about the cause of death but were frustrated with long delays waiting to obtain this. Many parents wanted more emotional support to be routinely provided. Most parents found the JHV helpful but a small minority of mothers found this intensely distressing. In comparison to JHVs, when police visited death scenes without paediatricians, information was missed and parents found these visits more upsetting. There were issues with uniformed non-specialist police traumatising parents by starting criminal investigations and preventing parents from accessing their home or collecting vital possessions.

**Conclusions:**

Overall most parents feel supported by professionals during the JAA; however there is scope for improvement. Paediatricians should ensure that parents are kept updated with the progress of the investigations. Some parents require more emotional support and professionals should assist them in accessing this.

**Electronic supplementary material:**

The online version of this article (doi:10.1186/s12887-017-0803-2) contains supplementary material, which is available to authorized users.

## Background

Many countries now have comprehensive child death review (CDR) processes with the aim of identifying the full reasons for each death to help prevent deaths in the future [1, 2]. CDR may involve prospective investigation of unexpected deaths; this might include physicians obtaining detailed medical histories from parents, analysis of death scenes by police and health care professionals, and multi-agency case reviews [3]. After a sudden child death, one of the parents’ greatest needs is to understand as fully as possible why their child died; many also want follow-up and emotional support from medical staff who cared for their child [4]. While responses to unexpected child deaths have developed rapidly over recent years, there has, to date, been relatively little research into parents’ experiences of these processes and, to our knowledge, there have been no qualitative data published on parents’ experiences of information provision, follow-up and support.

**Fig. 1:**
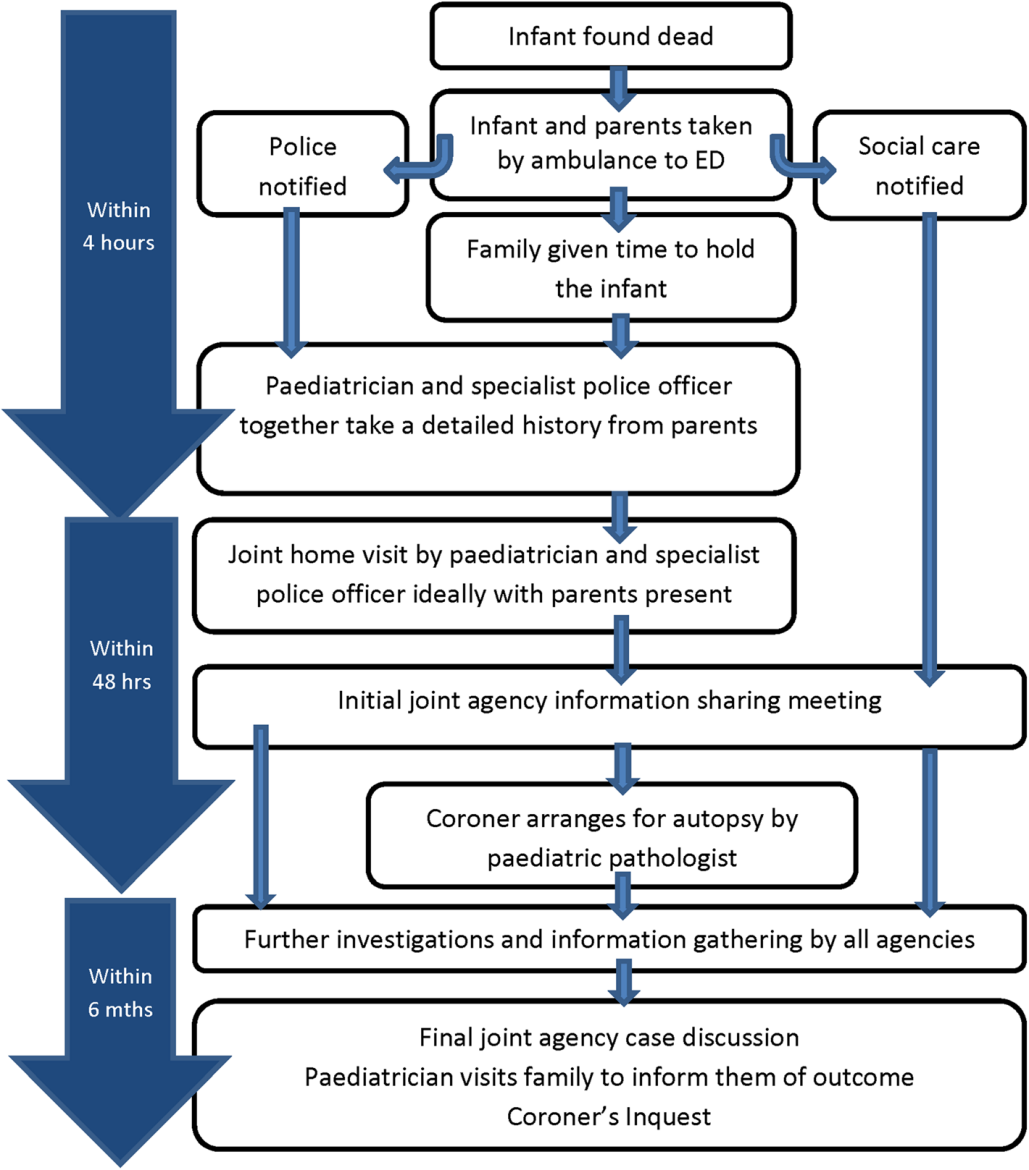
Flow chart of JAA investigation

Since 2008, England has adopted a joint agency approach (JAA) to unexpected child deaths. Police, health and social care jointly investigate deaths following national statutory guidance [5]. The stated aims of this JAA are to establish the complete cause of death and address the needs of family; these needs include those of emotional support, as well as potentially safeguarding other children. The JAA investigation is led by experienced paediatricians and the police response is provided by specialist teams with particular expertise in managing child death and child safeguarding enquiries. Key elements include taking the deceased infant to an Emergency Department, a paediatrician (possibly accompanied by the police) taking a detailed medical history from the parents, a joint examination of the scene of death by police and paediatrician, and follow-up for the parents. There is inter-agency communication throughout the JAA with a case conference to discuss the full causes of death. The process of the JAA is shown in Fig. 1. Despite statutory guidance, the practice of joint police and paediatric examination of the scene of death is variable and often police examine death scenes alone; however these cases are still considered to have had a JAA if there is inter-agency communication throughout the investigation.

The aim of this research project was to evaluate the JAA from the perspective of both bereaved parents and the professionals involved. This was as part of a larger mixed-methods study evaluating the joint agency response to sudden unexpected death in infancy (SUDI) [6]. Our research questions were:

What are the experiences of bereaved parents whose infants died suddenly and unexpectedly and were investigated by a JAA?

What are the experiences of professionals that relate to bereaved parents of using the JAA to investigate SUDI?

## Methods

The study used in-depth interviews with parents and professionals and documentary analysis of case records from all agencies. This enabled us to obtain further information on the process of investigations and parental support as well as allowing comparison between accounts from interviews and case records. We combined data from all sources into one analysis.

### Inclusion and exclusion criteria

We recruited parents of SUDI cases who had lived and died in the counties of Herefordshire, Shropshire, Staffordshire, Warwickshire, West Midlands and Worcestershire. Cases had to be aged between one week and one year at death and to have died between 01 September 2010 and 31 August 2013. We used the CESDI SUDI study definition of SUDI as being the death of an infant which was not anticipated as a significant possibility 24 h before the death or where there was a similarly unexpected collapse leading to or precipitating the events which led to the death [7]. We only recruited parents of cases where the JAA investigation was complete and excluded those with ongoing criminal investigations.

### Identification and recruitment of cases

The departments of pathology at Birmingham Women’s Hospital and Birmingham Children’s Hospital perform all SUDI post-mortem examinations for the region and they notified us of all eligible SUDI cases. Parents were approached through the local paediatrician and invited to participate in the study. Those who consented to the study were given the option of participating through an in-depth interview or a questionnaire, and were also asked to consent for the research team to access the case records from police, health, coroner and social care relating to the JAA investigation as well as access to their own primary care records. Participating parents gave written consent to the study having been fully informed of the study and of its risks and benefits.

### Social deprivation scores

We obtained the Income Deprivation Affecting Children Index (IDACI) [8] scores and ranks for all SUDI cases regardless of recruitment status; these were provided for us by the pathology department without disclosing any patient identifying information. This allowed us to compare social deprivation between recruited and non-recruited cases so that we could assess if the recruited sample was representative of SUDI cases more generally.

### In-depth parental interviews and questionnaires

The interviewer (JG) visited parents at home (or at a location of their choice) to conduct in-depth interviews between six and eighteen months after the death; this was their first contact with the interviewer. Parents could choose to have other friends and family present for support during the interview. Interviews were audio-recorded and field notes taken; the recordings were transcribed in full. We asked all parents who had completed an interview or questionnaire to take part in a follow-up interview around two years after the death; due to time constraints this was only possible for cases dying in the first two years of the study.

The interview covered the parents’ experiences of the JAA investigation from the time they discovered their baby had died until the final contact with professionals concerning the death. The follow-up interview covered the same issues, and parents were asked if they had had any further thoughts about the death or the JAA since the first interview. The interview schedule was developed with the advice of bereaved parents from the Lullaby Trust, the UK support group for SIDS parents; they recommended that we delayed interviewing parents for up to two years after the death to enable parents to reflect more on their experiences with professionals rather than on the grief of their loss.

Before the initial interview the interviewer was unaware of any case details so relied on listening to what parents thought was relevant to say. However, at follow-up interviews, the interviewer was able to probe parents further about the JAA, guided by analysis of initial interviews and case documents.

The questionnaires covered the same range of subjects as the in-depth interviews; parents either completed these with the interviewer during a visit or they were sent by post.

The interview schedules for both parental interviews and the professional interview can be seen in Additional file 1, and the parental questionnaire in Additional file 2.

JG is an experienced female SUDI paediatrician, conducting this research as part of a PhD; this was fully explained to all participants as part of the consent process.

### Case records

We studied infant health, police and social care records for details of events in the Emergency Department, investigations following the death and child safeguarding concerns; we also examined JAA final case discussion notes, post-mortem examination reports and coroners’ investigations for details of causes and risk factors for death. We asked general practitioners for a summary of all parents’ consultations for the year following the infant death. All data were extracted using standard proformae.

### In-depth professional interviews

After completion of in-depth parental interviews, JG interviewed the professionals who were involved in each JAA investigation. These interviews were conducted either in person or by telephone; they were audio-recorded, field notes taken and transcribed in full. Professionals were asked about their experiences of the JAA investigation specifically in relation to the recruited SUDI case, with questions guided by analysis of parents’ accounts.

### Qualitative data analysis

We analysed the qualitative data obtained from in-depth interviews, field notes and free text answers to questionnaires using a Framework Approach [9] with NVIVO 10 software. Data analysis was concurrent with interviewing. We checked all transcripts for accuracy with the audio-recording prior to coding. Starting with the parents’ interviews and associated field notes we undertook thematic analysis for their experiences of each stage of the JAA. After coding ten parental interviews the codes were summarised and discussed with the whole team and refined where needed. We managed the data from follow-up interviews identically to the data from initial interviews as they covered very similar subjects and no additional codes were needed for analysis of the follow-up interviews. The team included a wider study user group of SUDI professionals from all agencies and bereaved parents. We coded the professional interviews using the same coding structure. For each case we considered data from case records and professional interviews for corroboration and contrast with the parents’ data and created a framework matrix. The codes were developed into themes inductively; these related to different stages of the JAA investigation, positive and negative experiences, contact with primary care, and emotional support. Quotes have been given to illustrate each theme; they are only identified as being from mother, father or as a professional to ensure anonymity.

### Ethical issues

The initial contact with families was made through local paediatricians and parents were given time to consider whether or not to take part. Participation was on the basis of fully informed written consent for both parents and professionals; this included all participants giving consent for anonymised quotes to be used in research reports and publications. Parents were told that if they disclosed information that could lead to concerns about child abuse further action would need to be taken including possibly referring the matter to police and social care. Parents had the option to stop interviews or withdraw from the study at any time. After interviews, parents were provided with details of relevant support agencies.

The study received ethical approval from Solihull NHS Research Ethics Committee 12/WM/0211 and 10/H/1206/30.

## Results

### Recruitment

There were 113 SUDI cases during the time period of the study. Of these, 9 were ineligible as the JAA was incomplete or there were ongoing criminal investigations. Of the remaining 104 cases, 23 (22%) were recruited to the study; in 32 (31%) cases, paediatricians did not inform parents of the study; in 20 (19%), parents were lost to or did not want follow-up with the paediatricians; and in 29 (28%) parents were asked but declined to participate. The ethical requirement to warn parents about the interviewer’s duty to report possible child maltreatment did not deter parents from participating; no family declined the study after hearing this warning and two families had already had social care investigations as a result of the death.

We used the Income Deprivation Affecting Children Index (IDACI) [8] to compare social deprivation between recruited and non-recruited cases and there was no significant difference between these two groups of cases; these are shown in Tables 1 and 2.

**Table 1:** Comparison of Income Deprivation Affecting Children (IDACI) scores and ranks between recruited and no-recruited SUDI cases

	Recruited cases	Non recruited cases	Independent *t* test
Mean IDACI score	0.314	0.367	t (109) =−1.21 *p* = 0.229
95% CI limits of mean IDACI score	0.232–0.395	0.328–0.406	
Median IDACI rank	6702	5134	t (109) = 0.654 *p* = 0.514
Mean IDACI rank	9206	8012	
95% CI limits of mean IDACI rank	5617–12796	6419–9605

**Table 2:** Comparison of Income Deprivation Affecting Children (IDACI) ranks between recruited and non-recruited SUDI cases

	Recruited cases	Non recruited cases	Independent samples median test	Independent samples Mann – Whitney *U* test
Median IDACI rank	6702	5134	*p* = 0.446	*p* = 0.665

**Fig. 2:**
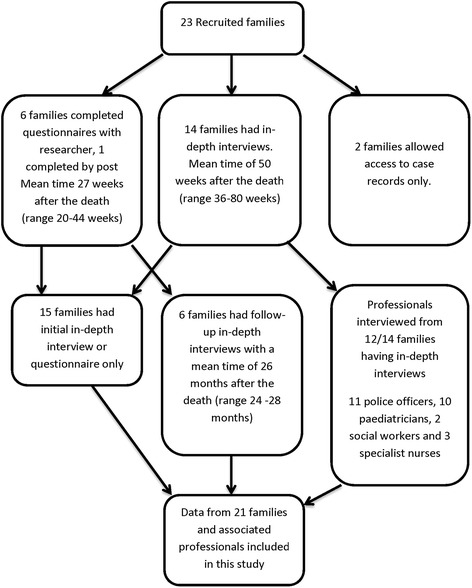
Flow chart of families’ and professionals’ participation in the study

The mean age at death of recruited cases was 100 days (95% CI 69–131 days). In 16 cases the death remained unexplained; being categorised as unascertained or having a diagnosis of SIDS, seven deaths were due to fully explained medical causes. The results in this paper are based on the 21/23 families having interviews or completing questionnaires and 26 interviewed professionals; two families opted for case note analysis alone so were not included in this qualitative study. Interviews lasted between one and four hours. Most parents were interviewed once between six and eighteen months after the death with some parents participating in follow-up interviews. The details of parental participation and data collection are shown in Fig. 2.

All parents appeared to vividly recall the events of their children’s deaths and their interaction with professionals despite the elapse of time; the Framework matrix enabled us to corroborate these accounts. Despite the low rates of recruitment, theoretical saturation of data was obtained, this was defined as the point when few new data emerged that were relevant to the developing theory [10].

### Results of qualitative analysis

There were three main themes relating to parents’ experiences: the JAA investigation itself, follow-up after the investigation, and emotional support throughout the process; within each theme parents described both positive and negative experiences. These themes were inter-related and linked to the aim of the JAA, which is to determine as far as possible the complete cause of death and to support the family. One further theme emerging from the data was a clear conflict between the requirements for professionals to thoroughly investigate deaths yet remain sensitive to the needs of bereaved families. There was no difference in the parental interview themes between cases with and without professional interviews.

### Overall experience of the JAA

Despite the immense trauma of the sudden death of their baby, parents in 14 families found the JAA a positive experience and four families a neutral experience. Parents were able to view the JAA positively and accept of the need for detailed investigation of their infant’s death as it was extremely important to them to know why their baby had died.

“Yes, I suppose I felt it was quite important to hear what the findings were really because it was unexpected, he was such a healthy boy and it was such a shock.......I really wanted to know and that was all really I guess.” (father)

For three families the JAA was a predominantly negative experience; these families found interacting with professionals when profoundly grief stricken very difficult. In all these families, this was compounded by the actions of the uniformed police who had commenced crime scene investigations at the home; this is discussed further later.

### The investigative process

#### The emergency department

The JAA investigation typically starts when the baby is brought by ambulance to the Emergency Department (ED). Most parents felt that they had been well cared for while in the ED, reflecting that while in hospital there are professionals, usually nurses, dedicated to caring for the parents, while other professionals are busy with the tasks of investigating the death.

“The nurse that was on duty that morning, she was just amazing. She even sat and cried with us …. So you know, they were lovely, but they helped us so much … they were fantastic.” (mother)

All parents who wished to were able to spend time holding their baby to say goodbye. Paediatricians and specialist police took detailed medical histories from parents who did not talk of finding this intrusive or distressing.

Most of the negative issues described related to isolated incidents in an otherwise overall good experience. Poor communication was at the root of most negative experiences. Some parents had found their infants cold, stiff and lifeless so correctly assumed that their baby was dead; they were then confused by reports from hospital staff that the baby was being resuscitated or to hurry to the hospital.

“But I was like ‘but she’s dead’ and she wouldn’t answer that question and so you have that moment of thinking ‘well maybe she’s not dead’. It was really horrible, absolutely awful.” (mother)

Only one family described their time in the ED very negatively; the mother was distraught as she wanted to see her baby again but the police had removed him contrary to the SUDI protocol. She only managed to see her baby after a two hour wait in the ED; during this time she felt uninformed and unsupported.

“…no-one had been applied to me sort of, to my care as such and we just didn’t know what was going on at any time…” (mother)

Professionals reported that the process in the ED seemed to work well for all the families except in the case of the mother described above.

#### Joint home visit by specialist police and paediatrician (JHV)

National statutory guidance on the management of SUDI stipulates that the scene of death should be examined jointly by specialist police and a paediatrician or specialist nurse, ideally with the parents present [5]. Joint home visits took place in 15/21 SUDI cases with the parents present for all these. In two cases, JHV were not necessary as the infant died outside of the home. In the remaining cases, specialist police conducted scene examinations without support from clinicians and in 2/4 cases in the absence of parents.

The JHV was a positive or neutral experience for most parents but for a few mothers it was a significantly negative experience. Many parents said that the JHV did not make their situation any worse; they accepted the need for it and were content just to get it done and then have some private family time. There were many different elements of JHVs that parents found helpful; these included providing information, support returning to the scene of death, understanding possible reasons why their baby may have died and showing compassion. Parents appreciated professionals who were non-judgemental and compassionate; often parents blamed themselves for the death at this time.

“Yeah, I never felt once like they were judging me or anything.” (mother)

“I think the practicalities as well of everything that comes after a death in the family, that them being able to do it so quickly afterwards is really good because then it was done, if I’m honest.” (mother)

“I always felt I should go back and say thank you to the police who attended.” (mother)

There were some issues with poor communication during JHVs. Some parents had to retell their version of events yet again at the JHV, some felt uncomfortable with detailed questioning and thought professionals lacked compassion. A minority of parents did not understand why a JHV was necessary.

“Well it felt uncomfortable because I felt…they kept just asking questions but you’re just upset and you don’t want to speak but they keep pushing and pushing.” (mother)

“I couldn’t understand why the doctors were here … why would they want to come and look at her bedroom? …The paediatrician was slightly…not rude but to the point … ‘did you have the heating on?’ … ‘I don’t know what day it is at the moment and no, the heating wasn’t on’.” (mother)

The JHV itself was hugely difficult for a small minority of mothers who were so distraught that they could not bear to talk to professionals at all and they could not face returning home to the scene of the death. The professionals were aware of how upsetting some mothers found the JHV and as a result reflected on how they could obtain the necessary information yet cause the minimum distress.

“I didn’t want to be there so…I walked out; I left my boyfriend in the house with the police and doctor…” (mother)

The professionals who took part in JHVs were overwhelmingly in favour of them, often stating that they were the most useful part of the JAA with seeing the sleep scene and general home environment proving invaluable. Police found it helpful for the SUDI paediatrician to take the lead in asking questions; they felt this reduced the parents’ anxieties about the police involvement.

“…So I think that works well …I wanted it to look like it’s a medical professional taking the lead here and we were there and supporting. I think the home visit is very good. Because you’ve got that…two different lenses really you know.” (police officer)

“I felt it went quite well…I would say that the police handled it very sensitively… But Mum was able to sort of demonstrate to us on the double bed exactly where the baby was, what position Mum was in, what position Dad was in…I think they found it helpful to do that, although distressing, as it is for all parents.” (specialist nurse)

Where police alone examined the scene of death, the parents were not always present and in those where they were, found these more upsetting than those carried out jointly with a paediatrician. For example, one father described:

“It felt like he was just checking everything in the house…you’re on pins by this stage anyway, your life is shit, it can’t get any worse than this and then you’ve got someone peering about your house like you’re a murderer.” (father)

In four of the cases with police home visits information about the sleep scene or medical history was missed or recorded inaccurately:

“…because I remember reading the report and thinking ‘well that’s not really right’, there were certain things that were slightly wrong…” (father)

“I mean we have been out [to the home] since then, but yes probably we did [miss details], we did on the sort of precise sleeping arrangements. Yes I’m sure we did.” (specialist nurse)

#### Social care

Although social care was involved in nearly all the cases, social workers only made direct contact with a minority of families. In some families the contact was mainly to offer support; these parents were very appreciative of the social care input. However in two families there were child safeguarding concerns and these parents felt misled in that they thought they were being offered support and only later realised their parenting was being assessed. The social workers in these cases described the difficulties they had in trying to explain their role to parents particularly as the safeguarding concerns did not arise immediately.

“To me that [the death] was just an excuse for the social workers to get involved, they wanted to be fully on me because there’s been domestic violence between me and the Dad.” (mother)

“… so I had kind of gone in and was genuinely trying to offer some support for Mum and the children, and I was talking about bereavement counselling and things like that … it was only when I picked up the case file … that I thought, there are too many other risk factors here that are going on.” (social worker)

#### The role of uniformed police

Ten families described particular issues with the actions of uniformed police officers furthering their distress; these events were all corroborated by police records. Uniformed officers had no prior training in managing SUDI cases, in contrast to the specialist police teams who are highly experienced in joint agency investigation of SUDI. Uniformed police often arrived at the home once the parents had telephoned for an ambulance; they seemed to treat the home as a crime scene and prioritised investigation of ‘the crime’ over supporting the parents. In five families, police refused parents access to collect vital possessions such as keys or mobile telephones and insisted families leave their homes immediately. In three cases, police officers did not allow parents to go to hospital with their infants, or removed infants from their parents while they waited for the ambulance to attend. All these actions were contrary to local multi-agency SUDI protocols.

“My wife went in the ambulance with the baby and my phone was upstairs in the bedroom and I needed my shoes as well; there was a police lady stood at the top of the stairs and she wouldn’t let me go upstairs….”(father)

“The ambulance just took him….. and the next thing the police were everywhere…. We said can we go and see him and they said no, we had to wait……but they just wouldn’t let us go….” (mother)

Although specialist police officers were available out of hours to manage SUDI cases, in four cases uniformed police waited until office hours to obtain specialist support. Sometimes specialist police were unaware of the actions undertaken by their uniformed colleagues but occasionally their actions were such that they negatively impacted on further investigations such as the analysis of the death scene.

“And I wasn’t sure whether the people [the uniformed police] that we were speaking to had had any experience of SUDIs or the SUDI protocol ….” (Specialist police officer)

“… So the police had gone in with great big size 10 boots and caused a lot of distress to the family, ahead of us getting there so … we had to recoup all of that…then it [the JHV] went quite well but we clearly could not look at properly the place where the baby had been sleeping because the police had removed all the bedding and so on was not how it had been.” (paediatrician)

A few families however, commented positively about uniformed police offering them emotional support and providing family members with transport to the hospital.

#### Follow-up for bereaved families – paediatricians and police

The parents’ experiences of follow-up were variable; they appreciated contact from paediatricians but found long waits for information difficult; most families waited nearly six months to learn of the cause of death. Half the families only had one follow-up visit from the paediatrician, primarily to explain the cause of death; these parents did not receive any information from the paediatrician or specialist nurse in the interim. The remaining families had telephone conversations or additional follow-up visits from the paediatrician or specialist nurse. Parents valued paediatricians telling them the cause of death in lay terms and having a chance to ask for more information; conversely those parents who first heard the cause of death at Inquest found this very distressing.

“The paediatrician was really good at this, how she read it to me; she was very clear and thorough. That I liked …. Them coming to your home and speaking to you before coroner’s court, I would absolutely agree with that…” (mother)

In eight families, parents felt that they had to do the chasing to get results; they often were telephoning the SUDI paediatrician or specialist nurse to be updated on the progress.

“…like they were supposed to keep in touch with me … just even if they never had any news… I don’t like the way it were done about that. I had to keep phoning and pestering them to know if there was anything….” (mother)

Only six families had follow-up contact with the police, usually by telephone. Most described this neutrally but these contacts caused significant distress to two families who found this intrusive and made them feel that they were under suspicion despite there being no child safeguarding concerns. However, many parents said that the police were sensitive in the way they returned any property to them following the investigation.

“My husband said the police officer was lovely. He took my husband in a room, they’d even put her clothes in a gift box and tissue paper inside, and they had even put a nappy in.” (mother)

#### Emotional support

Ten families felt let down by the lack of emotional support that was available and they often struggled to access bereavement services themselves. Some families found support from other bereaved parents, either through support groups or on an informal basis. The majority of parents found their family doctor to be helpful with many using them for bereavement support. In the year following the death mothers had a mean of 5.6 (95% CI 3.0–8.3) consultations for bereavement issues and fathers 3.3 (95% CI 0.2–6.8). Some parents however did not attend their family doctor at all and four parents’ primary care summary had no reference to the infant death. Often health visitors continued to visit mothers despite there being no pre-school children in the family, mothers valued their support enormously. Other mothers had no further contact with their health visitor and felt let down. However, not all parents wanted emotional support provision; many were content with the support given to them from their families and friends.

“I went to my doctor......I’m not so great on talking so they have sort of supported me because I haven’t actually been back to work or anything as such yet …” (mother)

“But then I got in touch with my friend’s health visitor, … She wasn’t my health visitor and I hadn’t got a baby anymore but she comes about every two weeks…. But she’s lovely.” (mother)

“I mean we went over it before…in hindsight, how pleased we were with the clinical side of things but disappointed with the mental health support.” (father)

Paediatricians and specialist nurses commented that they felt some families needed more emotional support than the JAA was able to provide.

## Discussion

Our findings show that it is possible to thoroughly investigate sudden infant deaths while being supportive and compassionate to families. The bereaved parents in this study valued the JAA; they particularly appreciated the detailed information on the cause of their child’s death but they felt let down by long waits for this information and the lack of emotional support that was provided. Most parents found the JHV helpful but a small minority of mothers found these intensely distressing. There were issues with uniformed police who had little knowledge of SUDI, increasing parents’ distress by starting criminal investigations and limiting parents’ access to their home and possessions. These actions made subsequent investigation by specialist police more challenging.

This study allowed for a very detailed understanding of cases due to the triangulation of data within each case from parental interviews, professional interviews and case records from every agency. We could confirm parents’ accounts that seemed questionable such as some of the actions of uniformed police. We captured a wide diversity of parent and professional experiences including both good and bad experiences, with recruited cases reflecting the social diversity of SUDI cases in the region as a whole. Given the diversity of experiences and theoretical saturation of data, the findings of this study are likely to be relevant to the management of sudden infant deaths in other locations with similar detailed investigative processes. A limitation of this study was the low recruitment; this occurred more commonly when there were long delays in the JAA process resulting in families being lost to follow-up prior to recruitment. An audit of JAA processes in part of the study area showed that only 64% of families were offered follow-up after SUDI [11]. This may mean that non-recruited families had significantly poorer experiences of the JAA than recruited ones.

Despite many countries having detailed child death investigative processes there are as yet few publications concerning parents’ experiences and none that we are aware of with qualitative data; this study is therefore unique. Parents in our study all found follow-up visits with the paediatrician helpful, similarly other studies of bereaved parents have found that parents would like more follow-up from doctors [12–15]. However, a recent US study of parents’ experiences of multi-disciplinary investigation of sudden child death found that one-third of parents had no follow-up contact at all with the teams investigating their child’s death and a further third only receiving the cause of death by post [16]. Parents may have fears relating to the survival of subsequently-born children [17]; more professional support and explanation around the time of death may help parents in the future. In addition, our study provides information about the benefits of joint death scene analysis by police and paediatricians with parents present: it was acceptable to most parents and professionals found it an extremely helpful way of working. In contrast, police analysis of death scenes alone led to missed information and greater parental distress. Similarly, a US review of SUDI cases showed the benefit of death scene analysis by an experienced health care professional compared to police officers alone [18] and a study of the JAA used within a SIDS research project found that specialist SUDI detectives were vital to ensure effective investigation and joint agency working [19].

## Conclusions

Our results provide reassurance to professionals that detailed child death investigations are acceptable to bereaved parents and provide them with valuable information as to why their child died. Despite the predominantly positive experiences of bereaved parents, the study raises a number of issues concerning how the management of SUDI could be improved. The actions of uniformed police caused considerable distress to many families; the police should consider how best to respond to SUDI and provide further training on SUDI for uniformed officers if they are to continue to be part of this response. All parents wanted to know why their baby died, most waited six months for this information. Ideally, parents should be kept up to date with the progress of investigations and informed of delays. Many parents wanted more emotional support to be provided by the JAA; child death review teams may wish to contemplate how they can assist parents to access appropriate bereavement support.

## Additional files

Additional file 1: In- depth interview guides. Parental in-depth interview guide. This is the interview guide used with all bereaved families during the first or only interview. Parental in-depth follow-up guide. This is the interview guide used with bereaved families who had a follow-up interview. Professional in-depth interview guide. This is the interview guide used with professionals. (DOCX 24 kb)
Additional file 2: Questionnaire. Parental questionnaire. This is the questionnaire completed by parents either during a visit from the interviewer or by post. (DOCX 71 kb)

## Abbreviations

CDR: Child Death Review
ED: Emergency Department
JAA: Joint Agency Approach
JHV: Joint Home Visit
SIDS: Sudden Infant Death Syndrome
SUDI: Sudden Unexpected Death in Infancy
