# Supplementary material for: Rigour and Rapport: a qualitative study of parents’ and professionals’ experiences of joint agency infant death investigation
Source: BMC Pediatr. 2017 Feb 7;17:48. doi: 10.1186/s12887-017-0803-2 (PMC5297208; doi:10.1186/s12887-017-0803-2)
Supplement: Additional file 1: — In- depth interview guides. Parental in-depth interview guide. This is the interview guide used with all bereaved families during the first or only interview. Parental in-depth follow-up guide. This is the interview guide used with bereaved families who had a follow-up interview. Professional in-depth interview guide. This is the interview guide used with professionals. (DOCX 24 kb) [file 12887_2017_803_MOESM1_ESM.docx]

# Appendix 4 In-depth interview schedules for parents’ and professionals’ interviews.

### WM SUDI study qualitative parental interview questions

### Infant study reference number…………………………………………..

| Name of Baby |  |
| --- | --- |
| Other names baby was known by |  |
| Date of Birth |  |
| Date of Death |  |
| Age at Death |  |
| Time between death and interview |  |

| Location of Interview |  |
| --- | --- |
| Date of Interview |  |
| Start time of interview |  |
| Finish time of interview |  |

| Names of those present at interview | Relationship to baby | Age |
| --- | --- | --- |

###

### Introductory questions:

1. Tell me about your pregnancy with *name*?

2. How was the birth, when did you go home?

3. What was *name* like as a baby?

### Events at the time of death:

1. What happened when *name* died?
2. What happened at the hospital?

### Home visit:

1. Who came to see where *name* died? Was it the police? Was a doctor with them?
2. What did they do in your home?
3. How did you feel about it at the time?
4. How do you feel about it now?
5. If there was anything different that the police or doctor could have done what would it be?
6. Is there anything else you want to tell me about the police and doctor’s visit?

### Follow-up:

1. Did the children’s doctor or other professional come and see you at home to discuss why *name* died?
2. Were you offered a hospital appointment to discuss this instead?
3. Would you have liked an appointment?
4. How did you feel about the follow-up visit or appointment?

### Knowledge of cause of death:

1. Can you explain to me what you understand of why *name* died?
2. Who explained about *name*’s death to you and when?

### Parental health following the death:

1. How would you describe your health after *name*’s death?
2. How is your health now?

### Fathers’ experiences:

1. How did you react to *name’s* death? Was this different to your partner’s reaction?
2. How did other people treat you? Was it different to your partner?

## WM SUDI Study follow-up qualitative parental interview schedule

Infant study reference number…………………………………………..

| Name of Baby |  |
| --- | --- |
| Other names baby was known by |  |
| Date of Birth |  |
| Date of Death |  |
| Age at Death |  |
| Time between death and interview |  |
| Date of original WMSUDI interview |  |

| Location of Interview |  |
| --- | --- |
| Date of Interview |  |
| Start time of interview |  |
| Finish time of interview |  |

| Names of those present at interview | Relationship to baby | Age |
| --- | --- | --- |
|  |  |  |
|  |  |  |
|  |  |  |

##### **Interview Guide**

#### Introductory Questions

1. How have things been since we last met? How are you now?

#### Changes in Childcare Practice (For families with new babies:)

2. How have you found caring for *new name*?

3. Are you doing anything differently to before?

#### Parental Wellbeing

4. How has your physical and emotional health been since we last met?

5. Have you may changes to your lifestyle or family life that you might not have made if *name* was still alive?

6. Do you think you have changed as a person as a result of *name's* death? How much of this change do you think is due to *name's* death?

#### Views on events of the Joint Agency Approach

7. Have your thoughts on what happened with the police, doctors, social workers or anyone else involved after s/he died changed?

8. Has the way you think about police, doctors or social workers changed as a result of *name's* death?

9. What is your understanding of why *name* died?

#### Use of support services

10. Have you been to any counselling or bereavement services? Did you find this helpful?

#### CDOP Process

11. Did you have any involvement with the Child Death Overview Panel? How did you find this process?

#### Alcohol and Tobacco

12. Can you tell me how much alcohol (if any) you drink each week at present?

13. Can you tell me how many cigarettes (if any) you smoke each week at present?

## West Midlands SUDI Study Professional interview schedule

| Name of Professional |  |
| --- | --- |
| Agency |  |
| Work base |  |
| Date of Interview |  |
| Reference number of infant case |  |

Professional Background

1. How long have you worked with SUDI cases for?
2. How many cases have you managed according to the joint agency approach?

*All the following questions refer to the case of baby reference number …… Please try to answer the questions without disclosing confidential case information.*

Police and Paediatricians only

1. Did you take part in a joint home visit? If not why not?
2. Did you find this joint home visit useful? If not why not?

All Agencies

1. Was there a final case discussion? Were you able to attend? If not why not?
2. Did you find the case discussion useful? If not why not?

Paediatricians only

1. Did you inform the family of the results of the case discussion? How? If not how were they informed?
2. How did you find this process?
3. Have you arranged further follow-up for the family?

All Agencies

1. How did you feel that the multi-agency process worked in this case?
2. What do you think were the most useful elements (if any) of the joint agency approach in investigating this case?
3. What do you think were the least useful elements (if any) of the joint agency approach in investigating this case?
4. Were there any particular difficulties with the joint agency approach in this case?
5. Is there anything that you would have wanted to have done differently in this case? Why?
6. Do you have any further views on the joint agency approach that you would like to share?
