# Supplementary material for: Rigour and Rapport: a qualitative study of parents’ and professionals’ experiences of joint agency infant death investigation
Source: BMC Pediatr. 2017 Feb 7;17:48. doi: 10.1186/s12887-017-0803-2 (PMC5297208; doi:10.1186/s12887-017-0803-2)
Supplement: Additional file 2: — Questionnaire. Parental questionnaire. This is the questionnaire completed by parents either during a visit from the interviewer or by post. (DOCX 71 kb) [file 12887_2017_803_MOESM2_ESM.docx]

# Appendix 6 Parental structured interview or self-completion questionnaire

Version 4 dated 10/11/11

| Name of baby |  |
| --- | --- |
| Date of Birth |  |

| Name of parent |  | |
| --- | --- | --- |
| Age of parent |  | |
| Date questionnaire completed |  | |
| Questionnaire completed by - please circle correct answer | mother | father |

##

### Please complete the questionnaire by yourself. There is one copy of the questionnaire for each parent.

**This questionnaire is about how you thought and felt about the professionals ( for example police, doctors, nurses, social workers) at the hospital and who might have visited you at home after your baby died**.

**1a Was your baby taken to hospital?**  Yes/No *please circle correct answer*

If no - where was your baby taken to? ....................................................................

*If your baby was not taken to hospital after they had died please go straight to question 2a.*

**1b Which hospital was your baby taken to?** .............................................................

**1c Did the ambulance or hospital staff try to resuscitate (do CPR/ heart massage) your baby**? Yes/No/Not sure *please circle correct answer*

**1d Did you have the opportunity to hold your baby after treatment had been stopped at the hospital?** Yes/No/Not sure *please circle correct answer*

**1e Using a scale of 1 -5 with 1 being far too little time, 3 being about right and 5 being far too much time, how much time would you have liked to have had to hold your baby after treatment had been stopped?**

1 2 3 4 5

far too little a bit little about right a bit much far too much

**1f Is there anything else you want to say about holding your baby in hospital after treatment had been stopped?** Please write it in the space below (you can continue on another piece of paper if needed)

...................................................................................................................................................................................................................................................................................................................................................................................................................................

**1g Did a children’s doctor (paediatrician) talk to you after your baby died?**

Yes/No/Not sure *please circle correct answer*

**1h Did the doctor ask you about....**

**Your baby's health?**  Yes/No/Not sure *please circle correct answer*

**The pregnancy and birth?** Yes/No/Not sure *please circle correct answer*

**What happened before you found your baby collapsed at home?**

Yes/No/Not sure *please circle correct answer*

**Other children at home?** Yes/No/Not sure *please circle correct answer*

**Anything else the doctor asked you about** - please write this down in the space below

**......................................................................................................................................**

**......................................................................................................................................**

**1j What did the doctor tell you?**

**About the post-mortem** Yes/No/Not sure *please circle correct answer*

**Possible reasons why your baby might have died**

Yes/No/Not sure *please circle correct answer*

**What would happen next, for example the police and doctor visiting you at home** Yes/No/Not sure *please circle correct answer*

Anything else you can remember that the doctor told you about - please write this down in the space below

**......................................................................................................................................**

**......................................................................................................................................**

**1k. Using a scale of 1 to 5, with 1 being very uncaring, 3 being neutral (neither caring or uncaring) and 5 being very caring:**

How caring do you think the hospital staff were towards you when your baby died?

*please circle the number to show how caring you think they were*

**1 2 3 4 5**

very uncaring a bit uncaring neutral a bit caring very caring

**1l. Using a scale of 1 to 5, with 1 being very uncaring, 3 being neutral (neither caring or uncaring) and 5 being very caring:**

How caring do you think the police at the hospital were towards you when your baby died? (There is another question later about the police who visited you at home)

*please circle the number to show how helpful you think they were*

**1 2 3 4 5**

very uncaring a bit uncaring neutral a bit caring very caring

**1m If there is anything else you want to say about your time in hospital please write it in the space below.**

**............................................................................................................................................................................................................................................................................**

**2a Did the police come and visit you at home and see where your baby died?**

Yes/No/Not sure *please circle correct answer*

**2b Did a specialist children’s doctor (paediatrician) or nurse visit you at home and see where your baby died?**

Yes/No/Not sure *please circle correct answer*

***2c* Did the specialist children’s doctor (or nurse) visit you with the police?**

*please circle correct answer*

Yes with police / Yes but not with the police / No /Not sure

**2d Did anyone else visit you with the police?**

Yes/No/Not sure *please circle correct answer*

**If yes was it the:** *please circle correct answer*

**Midwife/ Health Visitor/ Family Doctor/Someone else** - **please write below who**

..............................................................................................................................

**2e**  **How long after your baby’s death did the police visit?** *please circle correct answer*

**same day / next day/did not visit/ later - please write below how many days**

**..............................................................................**

**2f How long after your baby’s death did the specialist children’s doctor (or nurse) visit?**

*please circle correct answer*

**Same day/ next day / did not visit/later -please write below how many days**

......................................................................................................

**2g Did any other professional visit you at home about your baby’s death?**

*please circle correct answer*

**Midwife / Health Visitor/ Family Doctor / Coroner's Officer / other person/ no one**

**If other person please write who ...............................................................**

**2h How long after your baby’s death did the other professional visit?**

*please circle correct answer*

**Same day/ next day / did not visit/ later -please write below how many days**

......................................................................................................

*Question 3a-d are about the specialist children's doctor or nurse visiting you at home after your baby's death. If the specialist children's doctor or nurse did not visit you please go to question 4 a*

*These questions are about your experience at the time of the visit and now looking back.*

**3a. Using a scale of 1 to 5, with 1 being very unhelpful, 3 being neutral (neither helpful or unhelpful) and 5 being very helpful:**

How helpful did you find it, at the time, to have a specialist children’s doctor (paediatrician) or nurse visit you at home to talk about your baby's death?

*please circle the number to show how helpful you think they were*

**1 2 3 4 5**

very unhelpful a bit unhelpful neutral a bit helpful very helpful

**3b Using a scale of 1 to 4, with 1 being very intrusive and 4 being not intrusive at all:**

How intrusive did you find it, at the time, to have a specialist children’s doctor (paediatrician) or nurse visit you at home to talk about your baby's death?

*please circle the number to show how intrusive you think they were*

**1 2 3 4**

very intrusive quite intrusive a very little intrusive not intrusive at all

**3c.** This question is about how your feelings about the professionals visiting you at home may have changed over time.

**Using a scale of 1 to 5, with 1 being very unhelpful, 3 being neutral (neither helpful or unhelpful) and 5 being very helpful:**

Thinking about the specialist children’s doctor (paediatrician) or nurse visit now, how helpful did you find the visit?

*please circle the number to show how helpful you think they were*

**1 2 3 4 5**

very unhelpful a bit unhelpful neutral a bit helpful very helpful

**3d** This question is about how your feelings about the professionals visiting you at home may have changed over time.

**Using a scale of 1 to 4, with 1 being very intrusive and 4 being not intrusive at all:**

Thinking about the specialist children’s doctor (paediatrician) or nurse visit now, how intrusive did you find the visit?

*please circle the number to show how intrusive you think they were*

**1 2 3 4**

very intrusive quite intrusive a very little intrusive not intrusive at all

*Question 4a - b are about what you might have felt if a specialist children's doctor or nurse did not visit you at home after your baby's death. If a specialist children's doctor or nurse did visit you at home after your baby's death please go to question 5a.*

**4a Using a scale of 1 to 5, with 1 being very unhelpful, 3 being neutral (neither helpful or unhelpful) and 5 being very helpful:**

How helpful do you think it would be to have a specialist children’s doctor (paediatrician) or nurse visit you at home, after your baby's death?

*please circle the number to show how helpful you think it might be*

**1 2 3 4 5**

very unhelpful a bit unhelpful neutral a bit helpful very helpful

**4b** **Using a scale of 1 to 4, with 1 being very intrusive and 4 being not intrusive at all:**

How intrusive do you think it might be to have a specialist children’s doctor (paediatrician) or nurse visit you at home, after your baby's death?

*please circle the number to show how intrusive you think it might be*

**1 2 3 4**

very intrusive quite intrusive a very little intrusive not intrusive at all

*Questions 5a-d are about the police visiting you at home about your baby's death. If the police did not visit you at home after your baby's death please go straight to question 6a.*

*These questions are about your experience at the time of the visit and now looking back.*

**5a. Using a scale of 1 to 5, with 1 being very unhelpful, 3 being neutral (neither helpful or unhelpful) and 5 being very helpful:**

How helpful did you find it, at the time, to have the police visit you at home to talk about your baby's death?

*please circle the number to show how helpful you think they were*

**1 2 3 4 5**

very unhelpful a bit unhelpful neutral a bit helpful very helpful

**5b Using a scale of 1 to 4, with 1 being very intrusive and 4 being not intrusive at all:**

How intrusive did you find it, at the time, to have the police visit you at home to talk about your baby's death?

*please circle the number to show how intrusive you think they were*

**1 2 3 4**

very intrusive quite intrusive a very little intrusive not intrusive at all

**5c.** This question is about how your feelings about the professionals visiting you at home may have changed over time.

**Using a scale of 1 to 5, with 1 being very unhelpful, 3 being neutral (neither helpful or unhelpful) and 5 being very helpful:**

Thinking about the police visit now, how helpful did you find the police visit?

*please circle the number to show how helpful you think they were*

**1 2 3 4 5**

very unhelpful a bit unhelpful neutral a bit helpful very helpful

**5d** This question is about how your feelings about the professionals visiting you at home may have changed over time**.**

**Using a scale of 1 to 4, with 1 being very intrusive and 4 being not intrusive at all:**

Thinking about the police visit now, how intrusive did you find the police visit? *please circle the number to show how intrusive you think they were*

**1 2 3 4**

very intrusive quite intrusive a very little intrusive not intrusive at all

**6a Have any other professionals, for example the Coroner's Officer, Health Visitor, family doctor or midwife, visited you some days or weeks later to talk about your baby’s death?**

*please write down which professionals visited you*

....................................................................................................................................................

*If no other professionals visited you please go on to question 7a*

**For each professional (apart from police and specialist children's doctor or nurse) please say how helpful or intrusive you found them.**

**6b** Type of professional (midwife, coroner's officer etc) .........................................................

**Using a scale of 1 to 5, with 1 being very unhelpful, 3 being neutral (neither helpful or unhelpful) and 5 being very helpful:**

How helpful did you find the professional's visit?

*please circle the number to show how helpful you think they were*

**1 2 3 4 5**

very unhelpful a bit unhelpful neutral a bit helpful very helpful

**Using a scale of 1 to 4, with 1 being very intrusive and 4 being not intrusive at all:**

How intrusive did you find the professional's visit? *please circle the number to show how intrusive you think they were*

**1 2 3 4**

very intrusive quite intrusive a very little intrusive not intrusive at all

***If no other professionals visited you please go on to question 7a***

**6c** Type of professional (midwife, coroner's officer etc) **.........................................................**

**Using a scale of 1 to 5, with 1 being very unhelpful, 3 being neutral (neither helpful or unhelpful) and 5 being very helpful:**

How helpful did you find the professional's visit?

*please circle the number to show how helpful you think they were*

**1 2 3 4 5**

very unhelpful a bit unhelpful neutral a bit helpful very helpful

**Using a scale of 1 to 4, with 1 being very intrusive and 4 being not intrusive at all:**

How intrusive did you find the professional's visit? *please circle the number to show how intrusive you think they were*

**1 2 3 4**

very intrusive quite intrusive a very little intrusive not intrusive at all

***If no other professionals visited you please go on to question 7a***

**6d** Type of professional (midwife, coroner's officer , Health Visitor, etc) .........................................................

**Using a scale of 1 to 5, with 1 being very unhelpful, 3 being neutral (neither helpful or unhelpful) and 5 being very helpful:**

How helpful did you find the professional's visit?

*please circle the number to show how helpful you think they were*

**1 2 3 4 5**

very unhelpful a bit unhelpful neutral a bit helpful very helpful

**Using a scale of 1 to 4, with 1 being very intrusive and 4 being not intrusive at all:**

How intrusive did you find the professional's visit? *please circle the number to show how intrusive you think they were*

**1 2 3 4**

very intrusive quite intrusive a very little intrusive not intrusive at all

Questions 7a -e are about how much respect the professionals showed you when they came to visit you after your baby's death. Please answer for each professional who visited you.

**7a Using a scale of 1 to 5 with 1 being no respect at all, 3 being neutral (neither lack of respect or respectful) and 5 being very respectful:**

How respectful were the police when they visited?

**1 2 3 4 5**

no respect at all just a little respect neutral quite respectful very respectful

**If no more professionals visited please go to question 8a.**

**7b Using a scale of 1 to 5 with 1 being no respect at all, 3 being neutral (neither lack of respect or respectful) and 5 being very respectful:**

How respectful was the specialist children's doctor (or nurse) when they visited?

**1 2 3 4 5**

no respect at all just a little respect neutral quite respectful very respectful

**If no more professionals visited please go to question 8a.**

**7c** For any other professional who visited you about your baby's death. Please write down the type of professional (for example midwife) **........................................................................................................**

**Using a scale of 1 to 5 with 1 being no respect at all, 3 being neutral (neither lack of respect or respectful) and 5 being very respectful:**

**1 2 3 4 5**

no respect at all just a little respect neutral quite respectful very respectful

**If no more professionals visited please go to question 8a.**

**7d** For any other professional who visited you about your baby's death. Please write down the type of professional (for example midwife) **........................................................................................................**

**Using a scale of 1 to 5 with 1 being no respect at all, 3 being neutral (neither lack of respect or respectful) and 5 being very respectful:**

**1 2 3 4 5**

no respect at all just a little respect neutral quite respectful very respectful

**If no more professionals visited please go to question 8a.**

**7e** For any other professional who visited you about your baby's death. Please write down the type of professional (for example midwife) **........................................................................................................**

**Using a scale of 1 to 5 with 1 being no respect at all, 3 being neutral (neither lack of respect or respectful) and 5 being very respectful:**

**1 2 3 4 5**

no respect at all just a little respect neutral quite respectful very respectful

Questions 8a -e are about how much the professionals listened to what you had to say when they came to visit you after your baby's death. Please answer for each professional who visited you.

**8a Using a scale of 1 to 5 with 1 being not at all and 5 being very much:**

How much did the police listen to you when they visited?

**1 2 3 4 5**

not at all just a little moderately quite a lot very much

**If no more professionals visited please go to question 9a.**

**8b Using a scale of 1 to 5 with 1 being not at all and 5 being very much:**

How much did the specialist children's doctor (or nurse) listen to you when they visited?

**1 2 3 4 5**

not at all just a little moderately quite a lot very much

**If no more professionals visited please go to question 9a.**

**8c** For any other professional who visited you about your baby's death.

Please write down the type of professional (for example midwife) ........................................................................................................

**Using a scale of 1 to 5 with 1 being not at all and 5 being very much:**

How much did the professional listen to you when they visited?

**1 2 3 4 5**

not at all just a little moderately quite a lot very much

**If no more professionals visited please go to question 9a.**

**8d** For any other professional who visited you about your baby's death.

Please write down the type of professional (for example midwife) ........................................................................................................

**Using a scale of 1 to 5 with 1 being not at all and 5 being very much:**

How much did the professional listen to you when they visited?

**1 2 3 4 5**

not at all just a little moderately quite a lot very much

**If no more professionals visited please go to question 9**

**8e** For any other professional who visited you about your baby's death.

Please write down the type of professional (for example midwife) ........................................................................................................

**Using a scale of 1 to 5 with 1 being not at all and 5 being very much:**

How much did the professional listen to you when they visited?

**1 2 3 4 5**

not at all just a little moderately quite a lot very much

**9** If you have any other views about professionals visiting you at home to talk about your baby's death please write them down here (please continue on another piece of paper if you would like) ..........................................................................................................................................................................................................................................................................................................................................................................................................................................

**Questions 10a-c are about how much you understand of why your baby died.**

**10a Do you know why your baby died?**

*please circle answer*

Yes, quite clearly / I have some idea but I am not quite sure/ No, I have little idea

**10b** Please write down what you understand of why your baby died, for example, she died of a heart problem that had not been known about before.

**..................................................................................................................................................................................................................................................................................................................................................................................................................**

**10c Who explained to you what your baby died of?**

*please circle answer*

**Specialist children's doctor or nurse/ Family Doctor / Police / Coroner's Officer /Someone else / No one did**

If someone else explained why your baby died please write down who this was

............................................................................................................................................

Questions 11a- d are about the Coroner's inquest into your baby's death.

**11a Was there an inquest into your baby’s death? Yes/No/Not sure** *please circle answer*

**11b Did you attend the inquest?** **Yes/No**  *please circle answer*

**11c Do you know the outcome of the inquest? Yes/No/Not sure** *please circle answer*

**11d** If you know the outcome of the inquest please write it down

....................................................................................................................................

**11e** If there is anything else you want to say about the Coroner's inquest please write it down in the space below

**............................................................................................................................................................................................................................................................................**

Questions 12 a -c are about your health after your baby's death

**12a** Have you had any health problems yourself, after your baby's death? Health problems include mental health difficulties such as anxiety, panic attacks or depression, as well as physical health problems such as asthma and back pain.

**Yes/No**  *please circle answer*

**12b** Compared to your health before your baby died, is your health the same, better or worse?

**Same/Worse/Better** *please circle answer*

**12c** Please complete the table below with details of the health problems you have had since your baby's death. Some examples have been put in the table already to show you how to fill it in. (If you have had no health problems please go on to the next question)

| **Health problem** | **How long after your baby's death the health problem started** | **How long did health problem last** | **Any other comments** |
| --- | --- | --- | --- |
| *Anxiety attacks* | *Straight away* | *Still a problem* | *Seeing counsellor* |
| *Back pain* | *1 month* | *3 months* | *Had painkillers* |
| *Chest infection* | *3 months* | *2 weeks* | *Had antibiotics* |
|  |  |  |  |
|  |  |  |  |
|  |  |  |  |
|  |  |  |  |
|  |  |  |  |

Questions 13a -d are about your employment

**13a When your baby died were you:**

*please circle answer*

**in employment/ maternity leave/stay at home parent/ at college/ unemployed**

**13b Please write down the last job you had or if you were at college please write down your college course.**

**......................................................................................................................................**

**13c Have you returned to work or college since your baby's death?**

**Yes/No**  *please circle answer*

**13d If you returned to work or college, please write down when you returned.**

**....................................................................................................................................**

**13e Using a scale of 1 to 5, with 1 being very unhelpful, 3 being neutral (neither helpful or unhelpful) and 5 being very helpful:**

How helpful did you find your employer or college after your baby's death?

*please circle the number to show how helpful you think they were*

**1 2 3 4 5**

very unhelpful a bit unhelpful neutral a bit helpful very helpful

Questions 14a - are about smoking, alcohol and drug use

**14a Please write down how many cigarettes (if any) you smoke each day now**

**.................................................... .......................................**

**14b Please write down how many cigarettes (if any) you were smoking each day at the time your baby died**

**...................................................................................**

**14c Please write down how many units of alcohol you drink each week now.** (One unit of alcohol is a small glass of wine, half a pint of beer or lager or one measure of spirits.)

**................................................................................................................**

**14d Please write down how many units of alcohol you were drinking each week at the time your baby died.**

**........................................................................................................................**

**14 e Please give details of any illicit drugs that you use now**.(Illicit drugs are street drugs such as cannabis, heroin and cocaine.)

**......................................................................................................................**

**14f Please give details of any illicit drugs that you were using when your baby died.**

**................................................................................................................................**

**Please complete the following questions about how you are feeling now.**
